# Pregnant women’s use and attitude toward Mobile phone features for self-management

**DOI:** 10.1186/s12911-023-02172-w

**Published:** 2023-04-26

**Authors:** Ehsan Nabovati, Mehrdad Farzandipour, Zahra Vahedpoor, Hossein Akbari, Shima Anvari, Reihane Sharif, Farhad Fatehi

**Affiliations:** 1 Health Information Management Research Centre, Department of Health Information Management & Technology, School of Allied Health Professions, Kashan University of Medical Sciences, Kashan, Iran; 2 Department of Gynecology and Obstetrics, School of Medicine, Kashan University of Medical Sciences, Kashan, Iran; 3 Department of Biostatistics and Epidemiology, School of Health, Kashan University of Medical Sciences, Kashan, Iran; 4 Centre for Health Services Research, the University of Queensland, Brisbane, Australia

**Keywords:** Mobile Health, mHealth, Mobile phone, Attitude, Pregnancy, Self-management

## Abstract

**Background:**

This study aimed to examine the current use of mobile phones by pregnant women and their attitudes towards the use of a variety of prenatal care services through mHealth.

**Methods:**

This descriptive cross-sectional study was conducted in Iran in 2021. The study population included 168 pregnant women who referred to specialist obstetrics and gynecology clinic. The data collection tool was a questionnaire that included the demographics of the participants, their current mobile phone usage, and their attitudes toward mobile phone use for prenatal care services. The data were analyzed in SPSS with descriptive and analytical statistics.

**Results:**

The majority of participants (84.2%) had a smartphone and access to mobile internet. More than half of the respondents (58.9%) used their mobile phone for (only) phone calls, and 36.7% occasionally used mobile internet to access prenatal care services. To get information about the pregnancy and to communicate with other pregnant women, the participants mainly used social media, and to get reminders, they preferred phone calls.

**Conclusions:**

In this study, pregnant women have a positive attitude towards using mobile phones for obtaining health services and prefer social media to seek prenatal care services. There seems to be a need for pregnant women to have high levels of digital health literacy and be advised by healthcare providers on using this technology to access prenatal care services.

**Supplementary Information:**

The online version contains supplementary material available at 10.1186/s12911-023-02172-w.

## Background

Pregnant women are among the most vulnerable and at-risk groups due to pregnancy and childbirth complications. Millions of women worldwide suffer negative pregnancy experiences every year [1]. The lack of information during pregnancy is an important factor leading to adverse clinical outcomes for pregnant women, fetuses, and newborns [2]. Improving the knowledge of pregnant women, providing proper education, and appropriate care during pregnancy are important factors in preparing the mother, reducing medical interventions and consequently reducing adverse maternal, fetal and neonatal complications [3]. Pregnant women are often looking for information about their pregnancy and are willing to share their experiences with others. In the past, pregnant women received pregnancy information from others (e.g. midwives, physician, friends, or relatives). However, electronic media such as the internet and mobile phones now offer easy and convenient access to such information [4].

Mobile phones are now widely used as a convenient tool for delivering health support programs, referred to as mobile health (mHealth). mHealth is a component of electronic healthcare (eHealth), i.e. the use of mobile phones and wireless technology to improve healthcare delivery [5]. mHealth is now recognized as a useful tool for accessing health services, particularly in areas with limited health workforce, limited financial resources and high disease burden, such as in developing countries [6], and can also be considered as an effective intervention to improve care in low- and middle-income countries [7–9]. mHealth interventions provide the potential to improve pregnancy outcomes and maternal health by overcoming barriers of time and place [10].

Women of childbearing potential who are expecting a child are particularly frequent users of mHealth [11, 12] and primarily use the internet, social media and smartphone applications (apps) to obtain health information on a wide range of obstetric and pediatric topics [13]. Numerous studies have been conducted on the use of mHealth during pregnancy, which indicate the satisfaction and active participation of pregnant women in self-management activities [14–16]. A key component to the success of any mHealth system is positive user attitudes towards the use of this technology [17, 18]. To date, only a few studies have examined pregnant women’s attitudes towards using mobile phones for prenatal care [19–21]. A study by Waring et al. [19] in the United States reported that pregnant women are interested in using websites or smartphone apps to gain weight during pregnancy. Another study [20] found that most pregnant women were interested in receiving short message services (SMS) and phone calls with educational information about pregnancy and infant health.

To the best of our knowledge, no similar studies of pregnant women’s use of mobile phones and their attitudes towards the use of prenatal care have been conducted in developing countries. Previous studies in other countries have focused on pregnancy weight monitoring [19] and pregnancy-related services, but have not examined all mobile phone features [20, 22]. Considering the importance of educating pregnant women about appropriate prenatal care and the potential of mHealth to improve these activities, the present study aimed to examine the current use of mobile phone by pregnant women (e.g., phone calls, SMS, health-related mobile apps, web, email, and mobile social networks) and their attitude towards receiving a variety of prenatal care services through mHealth.

## Methods

### Study design and setting

This survey study was conducted in 2021 in the city of Kashan, Iran. The study population consisted of pregnant women attending a specialty and subspecialty obstetrics and gynecology clinic affiliated with Kashan University of Medical Sciences. This clinic with the most referrals is the only limited surgery center for women in Kashan and provides services such as midwifery, ultrasound, mammography, biopsy, radiology and operating room. Inclusion criteria were (A) a gestational age of two months or more, (B) possession of a mobile phone (either a simple phone or a smartphone), and (C) educational level of at least high school diploma. In a similar study by Cormick et al. [20], 95.9% of the pregnant women had a positive attitude towards receiving SMS. Using a 99% confidence interval and an accuracy of 0.05 in estimating the mean, the required minimum sample size was calculated to be 105, and with the availability of more samples, this increased to 168 pregnant women.

### Questionnaire

In order to develop a questionnaire for data collection, the relevant literature was reviewed and questionnaire items were combination of identified mobile phone features in a systematic review and the literature on technology use and adoption. Then a preliminary draft of the questionnaire was prepared according to the opinions of experts on pregnancy-related information (from obstetrics and gynecology specialists) and also on mobile phone features (from health information management and medical informatics experts). To determine the face validity, the questionnaire was first distributed and released to two experts in health information management and medical informatics. The content validity of the questionnaire was assessed by 12 experts (specialists in obstetrics and gynecology, health information management and medical informatics). The validity of each item (i.e. relevance and clarity) as well as the validity index of the tool as a whole (i.e. comprehensiveness) were determined using a Likert scale (from 1= ‘unfavorable’ to 4= ‘highly favorable’). Each item’s content validity was assessed based on the Content Validity Index (CVI), and if it was less than 0.7, the item was revised and modified. The Content Validity Ratio (CVR) was also determined based on the Lawshe’s table and items with values less than 0.56 were eliminated. The reliability of the questionnaire was determined using the split-half method and its Cronbach alpha was calculated at 0.87.

After validating the data collection tool, the resulting questionnaire had 46 items in three main sections: demographic information (11 items), current mobile phone use (22 items), and attitudes towards mobile phone use for prenatal care services (13 items). Five of these items were yes/no questions. A “yes” answer scored one point and a “no” answer zero points, and six items were four-choice questions, with ‘everyday’ =1 point, ‘several times per week’ =0.6, ‘occasionally’ =0.3, and ‘never’ =0. The items on the pregnant women’s use (12 items) and attitude to use (12 items) were rated in such a way that use or attitude to use each feature was rated with one point, and no use or no attitude to use was rated with zero points. The sum of the scores from the two sections of the questionnaire (i.e. use of and attitude towards use) were divided into three groups based on the 25th and 75th percentile as low, medium or high.

### Data collection

Participants were selected using a simple (convenience) sampling method. A researcher (SA) visited the obstetrics and gynecology clinic and distributed the questionnaires to eligible pregnant women and asked those willing to participate to complete the questionnaire. The purpose of the study was then explained to all users and they signed the written informed consent form. Identifiable personal information was not collected; all data were kept confidential and safe.

### Statistical analysis

Data were analyzed in Statistical Package for the Social Sciences (SPSS) 22 (IBM SPSS Statistics, IBM Corporation, Armonk, NY, USA). First, the number and frequency of the demographic variables, accessibility and use of mobile phone were calculated. Then the mean and the standard deviation (SD) of the quantitative variables as well as the frequency of the use and the use attitude of the pregnant women in relation to the demographic variables were calculated. Then the sum of the scores of the two sections (i.e. usage and attitude to use) was calculated and based on the 25th and 75th percentile the participants were divided into three groups according to their usage rate: ‘low’ for scores < 15.52, ‘moderate’ for scores of 15.52–20.27 and ‘high’ for scores > 20.27. With regard to their attitude to use, they were classified as ‘low’ for scores < 13, ‘moderate’ for scores of 13-14.25, and ‘high’ for scores of > 14.25. The frequency percentage was also calculated for each of the items. The relationships between the demographic variables and the questionnaire sections (i.e., usage and attitude towards usage) were assessed using the chi-square test or Fisher’s exact test. The threshold probability of P < 0.05 was assumed as the statistical significance level.

## Results

### Participants’ characteristics

Of the 168 questionnaires distributed, 158 (94%) were completed. The mean age of the participants was 28.39 ± 5.84 years. A total of 93 pregnant women (58.9%) had less than bachelors education and 152 (96.2%) lived in the city. Most of the participants (89.9%) referred to a physician for prenatal care services, and 79.1% of them were not employed. The mean gestational age was 5.58 ± 2.29 months, and 51.9% of them were the first pregnancy (Table 1).

**Table 1:** Participants’ demographic information (N = 158)

Demographic variable		Number (%)
**Age (years)**	≤ 25	52(32.9)
	> 25	106 (67.1)
	**Mean ± SD**	28.39 ± 5.84
**Education level**	Less than bachelor’s degree	93(58.9)
	Higher than bachelor’s degree	65(41.1)
**Area of residence**	City	152(96.2)
	Village	6(3.8)
**Under the supervision**	Physician	142(89.9)
	Midwife	5(3.2)
	Health Center	7(4.4)
	Without supervision	4(2.5)
**Nationality**	Iranian	152(96.2)
	Non-Iranian	6(3.8)
**Employed**	Yes	33(29.9)
	No	125(79.1)
**Pregnancy month**	5 and less	76(48.1)
	6 and above	82(51.9)
	**Mean ± SD**	2.29 ± 5.58
**Previous pregnancy**	Yes	74(48.1)
	No	82(51.9)

Table 2 shows the general findings regarding the use of and attitudes towards the use of mobile phones by pregnant women to access prenatal care services. Of the 158 participants, 131 (82.9%) used mobile phones to obtain pregnancy-related services. 133 (84.2%) participants had smartphones and access to mobile internet. 60 (38%) of the participants had at least one pregnancy-related application (app) on their smartphone. A total of 122 pregnant women (72.2%) had a positive attitude in using mobile phones to receive prenatal care services.

**Table 2:** Use and attitude to use mobile phone (N = 158)

Item	Number (%)
Use of mobile phones to receive prenatal care services	131 (82.9)
Mobile internet access	133 (84.2)
Have smartphone (possibility to install apps)	133 (84.2)
Installing mobile apps related to pregnancy	60 (38)
Attitude to use mobile phones to receive prenatal care services	122 (72.2)

Table 3 shows the use of mobile phones by pregnant women to obtain pregnancy-related information in four categories: every day, several times a week, sometimes and never. Of the 158 participants, 93 (58.9%) sometimes used phone calls to get pregnancy information (from friends, relatives, physicians, nurses and midwives), and more than half of them (54.4%) never used SMS, to get information. Participants sometimes used their internet (36.7%) and social media (35.4%) to obtain and access pregnancy information. Most participants never used mobile email to communicate with others (79.1%) and smartphone apps (58.9%) to access pregnancy information.

**Table 3:** The frequency of use of mobile phone by the pregnant women (n = 158)

Items	Number (%)			
	Everyday	Several Times per Week	Sometimes	Never
Receiving pregnancy-related information through phone calls (from friends, relatives, physicians, nurses and midwives)	7 (4.4)	18 (11.4)	93 (58.9)	40 (25.3)
Receiving pregnancy-related information through SMS* (from friends, relatives, physicians, nurses and midwives)	3 (1.9)	9 (5.7)	60 (38)	86 (54.4)
Using mobile Internet to obtain pregnancy-related information	27 (17.1)	37 (23.4)	58 (36.7)	36 (22.8)
Using social media (such as Telegram channels) to access pregnancy-related information	31 (19.6)	16 (10.1)	55 (34.8)	56 (35.4)
Using mobile email to communicate with others (friends, relatives, physicians, nurses and midwives) to receive pregnancy-related information	1 (0.6)	7 (4.4)	25 (15.8)	125 (79.1)
Using smartphone applications (software) to access pregnancy-related information	10 (6.3)	20 (12.7)	35 (22.2)	93 (58.9)

*SMS: Short Message Service

The results showed that pregnant women most frequently used internet searches to find information about avoiding harmful medications during pregnancy (42.4%), physiological changes during pregnancy (36.1%), pregnancy complications and types of delivery (35.4%), and nutritional needs during pregnancy (34.2%). In addition, participants had used phone calls the most to communicate with other pregnant women (21.5%) and to get reminders about appointments (20.9%), ultrasound scans (16.5%), and lab tests (15.8%). Meanwhile, the pregnant women had a greater attitude towards using social media to get information about nutrition needs during pregnancy (35.5%), exercise and fitness (32.3%) and to communicate with other pregnant women (29.1%). They also had greater attitudes toward using phone calls to be reminded of appointments (19.6%), having ultrasound scans (19%) conducting lab tests (18.4%), and taking medications and supplements (17.7%). An additional file shows the frequency of use and attitude towards the use of mobile phones for the utilization of prenatal care services [Additional file 1].

The results showed that educational level, employment status and number of previous pregnancies had an impact on mobile phone use and attitude among pregnant women. This study found that pregnant women’s use was higher and their attitude was more positive in those with higher education (P < 0.004). Housewives also used mobile phones most often (P < 0.042), but their attitudes did not differ significantly from that of the others (P < 0.216). Mobile phone use among pregnant women who had experienced a previous pregnancy was significantly higher than the others (P < 0.001) and they also had better attitudes towards mobile phone use (P < 0.005). An additional file shows the frequency of use and attitude toward mobile phone use among pregnant women in terms of demographic details [Additional file 2].

## Discussion

### Principal findings

The majority of the participants in this study had a smartphone and had access to the internet via their mobile phones. More than half of those surveyed sometimes used phone calls and mobile internet to obtain pregnancy-related information. A significant number of participants never used both email and smartphone apps to receive pregnancy-related information. Pregnant women used the internet most often to find information about taking harmful medications during pregnancy, pregnancy complications and delivery methods, fetal development, and nutritional needs during pregnancy. Most participants used phone calls to communicate with other pregnant women and to receive reminders about appointments, taking medications and supplements, and laboratory tests and ultrasound scans. To get information about the pregnancy and to communicate with other pregnant women, the pregnant women were most interested in using social media and also to get reminders, they were most interested in using phone calls.

The results showed that a significant number of pregnant women had access to the internet through their mobile phone, consistent with the results of similar studies in other countries [13, 23]. Although physicians inform pregnant women about pregnancy during their visit to the clinic [24], they still need more information about pregnancy to boost their confidence. Therefore, to meet these needs, they use the internet as a source of information before a prenatal visit or immediately after a visit [25]. An Italian study by De Santis et al. [23] stated that using the internet is the easiest and quickest way to raise awareness and address concerns during pregnancy. Given the widespread use of the internet by pregnant women, there is a need to identify their information needs and provide them with useful information through the internet. Therefore, it is recommended that obstetricians and gynecologists participate in the design of content for digital resources (e.g. websites and smartphone apps) and present valid sources of information to pregnant women.

Based on the results of this study, the most common uses of internet searches by pregnant women were to obtain information about harmful medications during pregnancy, pregnancy complications and delivery methods, fetal development, and nutritional needs during pregnancy. These results are similar to those found in other studies of the most important information pregnant women look for on the internet [13, 26, 27]. In addition, most participants in this study used phone calls to communicate with other pregnant women and to receive reminders about appointments, medication and supplement intake, and laboratory tests and ultrasound scans. Similarly, a recent study by Yamin et al. in 2018 [22] indicated that most pregnant women in Afghanistan preferred phone calls over SMS for health information and medication reminders. This study found that Afghan women’s low literacy level was the reason behind their preference for phone calls. As the mobile phone is an inexpensive and easily accessible information tool, it can be used to counsel patients with chronic diseases as well as those who need continuous care in their self-management, such as in the case of pregnant women. However, it should be noted that some studies have recognized that phone calls follow-up is time-consuming [28, 29] and that pregnant women may not always be able to take phone calls due to their unique conditions.

The results showed that more than half of the respondents sometimes used mobile phone calls, and more than a third of them used mobile internet to get pregnancy-related information. Similarly, several studies have indicated that pregnant women search the internet at least once a month [13, 26, 30, 31]. Larson et al. [32] also reported that in Sweden, a higher frequency of pregnancy-related information is searched over the internet, ranging from once a month to 62 times a month. Additionally, a significant number of pregnant women in this study never used email and smartphone apps for pregnancy information. These findings contradict the results of several studies on the use of smartphone apps by pregnant women in other countries [16, 33, 34]. As noted in these studies, pregnant women frequently used pregnancy related smartphone apps and also reported that the apps are an important source of information and a tool for monitoring pregnancy. Trrip et al. [35] emphasized that in Australia, smartphone apps as self-monitoring tools have good potential to change pregnant women’s self-management behaviors, as they can improve access to educational sources, enhance face-to-face interaction and overcome some of the barriers associated with traditional healthcare (face-to-face counseling). Despite these advantages, Burt et al. [36] compared the most popular pregnancy apps and reported that their sources of information were not valid. Several studies have also reported that pregnant women were keen to consult a specialist instead of using smartphone apps [4, 15, 37]. In the present study, the low use by pregnant women may be due to a lack of knowledge about this new technology or a lack of trust in the information content of existing apps. As smartphone apps can be an important source of information for pregnant women, the involvement of healthcare professionals in their development, implementation and support is essential.

In the present study, pregnant women were most interested in using mobile social media to obtain information and communicate with other pregnant women and were also most interested in using phone calls for a variety of reminders. These results were consistent with the results of other similar studies. Kraschnewski et al. [38] reported that more than 80% of pregnant women used social media to obtain pregnancy information. Asiodu et al. [39] found that American pregnant women used social media as a practical, convenient, and valuable way to obtain prenatal information. Studies on social media have shown that these tools are one of the most important and important sources of health information [40, 41]. Pregnant women who have used these tools can share health and pregnancy information with similar people and groups and also communicate with other pregnant women [42]. While social media and mobile phones provide access to information easily, cheaply and without space and time constraints, the validity, content of the information and the quality of the information provided in these information sources must also be considered.

The findings showed that educational level, employment status and number of previous pregnancies had an impact on mobile phone use and attitude among pregnant women. Similarly, Shieh et al. [43] reported that educational level, work status, and number of pregnancies had a significant impact on internet use and access among pregnant women. A review showed that highly educated pregnant women found online health information to be reliable and useful, and significantly improved their confidence in making decisions about their pregnancy after using the internet [44]. A study by Song et al. in the US [45] demonstrated that the internet was not widely used by pregnant women with low income and low education. This may be because some pregnant women do not have access to the internet or a digital tool, nor are they able to search for health information online. Consequently, it can be said that sociodemographic factors are usually not the real factor behind adoption decisions but rather other underlying causes.

### Strengths and Limitations

Based on the literature review, this study is the first to be conducted in a developing country on the use of pregnant women and their attitudes towards the use of mobile phones for prenatal care. The results can be used as a basis for future studies on the design and development of mHealth interventions for pregnant women. The present study included a sample of 158 pregnant women in one city, which can be considered a limitation. In this study, all participants were selected from a medical center, however, the clinic had the highest number of referrals in Kashan, but the results may not be generalizable to the entire population of pregnant women in Iran.

### Implications for practice & Future Research

Given the evidence supporting pregnant women’s attitudes towards using mobile phones to seek self-management services, the following recommendations are proposed to improve uptake of these tools. Obstetrics and gynecology specialists take into account pregnant women’s concerns about the quality of information provided via mobile phones and also assure them of the benefits of using these tools. In this study, pregnant women may hardly use smartphone apps due to the lack of awareness of their advantages and lack of confidence in their information content, therefore collaboration of healthcare professionals for the design, development and implementation of these apps appears necessary. This study identified pregnant women’s information needs and their preferred types of features in order to receive prenatal care services, making designing future interventions based on pregnant women’s needs imperative. Further studies are needed to investigate the use and attitudes of pregnant women towards using mHealth technology in larger populations.

## Conclusion

Currently, in Iran as a developing country, pregnant women use internet search to get information about harmful medications, pregnancy complications and delivery methods, fetal development and nutritional needs during pregnancy. They use the phone call to communicate and receive reminders with other pregnant women. They also have positive attitudes towards using social media to seek prenatal care services. Therefore, there seems to be a need to examine the information content of digital resources from healthcare providers and to guide pregnant women in using these tools to access prenatal care services.

## Electronic supplementary material

Below is the link to the electronic supplementary material.

Supplementary Material 1

## Data Availability

The datasets generated and/or analysed during the current study are not publicly available (due to compromising the privacy of individuals) but are available from the corresponding author on reasonable request.

## Abbreviations

mHealth: Mobile Health
eHealth: Electronic Health
Apps: Applications
SMS: Short Text Message
CVI: Content Validity Index
CVR: Content Validity Ratio
SPSS: Statistical Package for The Social Sciences

## References

[1] UNCsF: United Nations Children’s Fund (UNICEF). The state of the World’s children 2009: maternal and newborn health. UNICEF; 2008.

[2] Blehar MC, Spong C, Grady C, Goldkind SF, Sahin L, Clayton JA (2013). Enrolling pregnant women: issues in clinical research. Women’s Health Issues.

[3] World Health Organizatio. WHO recommendations on antenatal care for a positive pregnancy experience. World Health Organization; 2016. 28079998

[4] Fleming SE, Vandermause R, Shaw M. First-time mothers preparing for birthing in an electronic world: internet and mobile phone technology.Journal of Reproductive and Infant Psychology. 32(3):240–53.doi: 10.1080/02646838.2014.886104.

[5] Martinez PR. A Qualitative Study on Patient Perceptions Towards mHealth Technology Among High Risk, Chronic Disease Patients 2015.

[6] Mechael P, Batavia H, Kaonga N, Searle S, Kwan A, Goldberger A (2010). Barriers and gaps affecting mHealth in low and middle income countries: policy white paper: Columbia university. Earth institute.

[7] Willcox JC, van der Pligt P, Ball K, Wilkinson SA, Lappas M, McCarthy EA (2015). Views of women and health professionals on mHealth lifestyle interventions in pregnancy: a qualitative investigation. JMIR mHealth and uHealth.

[8] Noordam AC, Kuepper BM, Stekelenburg J, Milen A (2011). Improvement of maternal health services through the use of mobile phones. Tropical Med Int Health.

[9] Lund S, Hemed M. Wired mothers: use of mobile phones to improve maternal and neonatal health in Zanzibar: University of Copenhagen. Danish research network for health (Enreca health); 2010.

[10] Tamrat T, Kachnowski S (2012). Special delivery: an analysis of mHealth in maternal and newborn health programs and their outcomes around the world. Matern Child Health J.

[11] Lima-Pereira P, Bermúdez‐Tamayo C, Jasienska G. Use of the Internet as a source of health information amongst participants of antenatal classes. Journal of clinical nursing. 2012;21(3‐4):322 – 30. doi:10.1111/j.1365-2702.2011.03910. x. 10.1111/j.1365-2702.2011.03910.x 22093043

[12] Lorence D, Park H (2006). Web-based consumer health information: public access, digital division, and remainders. Medscape Gen Med.

[13] Gao L-l, Larsson M, Luo S-y (2013). Internet use by chinese women seeking pregnancy-related information. Midwifery.

[14] Wallwiener S, Müller M, Doster A, Laserer W, Reck C, Pauluschke-Fröhlich J (2016). Pregnancy eHealth and mHealth: user proportions and characteristics of pregnant women using web-based information sources—a cross-sectional study. Arch Gynecol Obstet.

[15] Rodger D, Skuse A, Wilmore M, Humphreys S, Dalton J, Flabouris M (2013). Pregnant womena’s use of information and communications technologies to access pregnancy-related health information in South Australia. Aust J Prim Health.

[16] Lupton D, Pedersen S (2016). An australian survey of women’s use of pregnancy and parenting apps. Women Birth.

[17] Jenssen BP, Mitra N, Shah A, Wan F, Grande D. Using digital technology to engage and communicate with patients: a survey of patient attitudes. Journal of general internal medicine. 2016;31(1):85–92. doi.10.1007/s11606-015-3517. 10.1007/s11606-015-3517-x PMC4699992 26385117

[18] McGillicuddy JW, Weiland AK, Frenzel RM, Mueller M, Brunner-Jackson BM, Taber DJ (2013). Patient attitudes toward mobile phone-based health monitoring: questionnaire study among kidney transplant recipients. J Med Internet Res.

[19] Waring ME, Simas TAM, Xiao RS, Lombardini LM, Allison JJ, Rosal MC et al. Pregnant women’s interest in a website or mobile application for healthy gestational weight gain. Sexual & Reproductive Healthcare. 2014;5(4):182-4. doi.10.1016/j.srhc.2014.05.002. 10.1016/j.srhc.2014.05.002 PMC4250573 25433828

[20] Cormick G, Kim NA, Rodgers A, Gibbons L, Buekens PM, Belizán JM et al. Interest of pregnant women in the use of SMS (short message service) text messages for the improvement of perinatal and postnatal care. Reproductive health. 2012;9(1):9. doi.10.1186/1742-4755-9-9. 10.1186/1742-4755-9-9 PMC3453517 22866753

[21] Goetz M, Müller M, Matthies LM, Hansen J, Doster A, Szabo A et al. Perceptions of patient engagement applications during pregnancy: a qualitative assessment of the patient’s perspective. JMIR mHealth and uHealth. 2017;5(5): e73. doi.10.2196/mhealth.7040. 10.2196/mhealth.7040 PMC5466700 28550005

[22] Yamin F, Kaewkungwal J, Singhasivanon P, Lawpoolsri S. Women Perceptions of Using Mobile Phones for Maternal and Child Health Support in Afghanistan: Cross-Sectional Survey. JMIR mHealth and uHealth. 2016;6(4): e76. doi.10.2196/mhealth.9504. 10.2196/mhealth.9504 PMC5915672 29636317

[23] De Santis M, De Luca C, Quattrocchi T, Visconti D, Cesari E, Mappa I et al. Use of the Internet by women seeking information about potentially teratogenic agents. European Journal of obstetrics & gynecology and reproductive biology. 2010;151(2): 154–157. doi.10.1016/j.ejogrb.2010.04.018. 10.1016/j.ejogrb.2010.04.018 20478650

[24] Diaz JA, Griffith RA, Ng JJ, Reinert SE, Friedmann PD, Moulton AW (2002). Patients’ use of the internet for medical information. J Gen Intern Med.

[25] Huberty J, Dinkel D, Beets MW, Coleman J. Describing the use of the internet for health, physical activity, and nutrition information in pregnant women. Maternal and child health journal.2013;17(8):1363-72. doi.10.1007/s10995-012-1160-2. 10.1007/s10995-012-1160-2 23090284

[26] Kavlak O, Atan ŞÜ, Güleç D, Öztürk R, Atay N. Pregnant women’s use of the internet in relation to their pregnancy in Izmir, Turkey. Informatics for health and social care. 2012;37(4):253 – 63. doi.10.3109/17538157.2012.710686. 10.3109/17538157.2012.710686 22958087

[27] Luyben AG, Fleming VEM. Women’s needs from antenatal care in three European countries. Midwifery. 2005;21(3):212 – 23. doi.10.1016/j.midw.2004.11.001. 10.1016/j.midw.2004.11.001 15967548

[28] Kim H, Oh JA. Adherence to diabetes control recommendations: impact of nurse telephone calls. Journal of advanced nursing. 2003;44(3):256 – 61. doi.10.1046/j.1365-2648.2003.02800.x. 10.1046/j.1365-2648.2003.02800.x 14641395

[29] Dewar A, Scott J, Muir J. Telephone follow-up for day surgery patients: patient perceptions and nurses’ experiences. Journal of PeriAnesthesia Nursing. 2004;19(4):234 – 41. doi.10.1016/j.jopan.2004.04.004. 10.1016/j.jopan.2004.04.004 15293174

[30] Bakhireva LN, Young BN, Dalen J, Phelan ST, Rayburn WF (2011). Patient utilization of information sources about safety of medications during pregnancy. J Reprod Med.

[31] Lagan BM, Sinclair M, George Kernohan W. Internet use in pregnancy informs women’s decision making: a web-based survey. Birth. 2010;37(2):106 – 15. doi.10.1111/j.1523-536X.2010.00390. x. 10.1111/j.1523-536X.2010.00390.x 20557533

[32] Larsson M. A descriptive study of the use of the Internet by women seeking pregnancy-related information. Midwifery. 2009;25(1):14–20. doi.10.1016/j.midw.2007.01.010. 10.1016/j.midw.2007.01.010 17408822

[33] Lee Y, Moon M. Utilization and content evaluation of mobile applications for pregnancy, birth, and child care. Healthcare informatics research. 2016;22(2): 73–80. doi.10.4258/hir.2016.22.2.73. 10.4258/hir.2016.22.2.73 PMC4871848 27200216

[34] O’Higgins A, Murphy OC, Egan A, Mullaney L, Sheehan S, Turner M (2014). The use of digital media by women using the maternity services in a developed country. Ir Med J.

[35] Tripp N, Hainey K, Liu A, Poulton A, Peek M, Kim J et al. An emerging model of maternity care: smartphone, midwife, doctor? Women and Birth. 2014;27(1): 64–67. doi.10.1016/j.wombi.2013.11.001. 10.1016/j.wombi.2013.11.001 24295598

[36] Bert F, Passi S, Scaioli G, Gualano MR, Siliquini R. There comes a baby! What should I do? Smartphones’ pregnancy-related applications: A web-based overview. Health informatics journal. 2016;22(3):608 – 17. doi.10.1177/1460458215574120 10.1177/1460458215574120 25900813

[37] Hearn L, Miller M, Lester L. Reaching perinatal women online: The Healthy You, Healthy Baby website and app. Journal of Obesity. 2014; 2014:573928. doi.10.1155/2014/573928 10.1155/2014/573928 PMC4020447 24872891

[38] Kraschnewski JL, Chuang CH, Poole ES, Peyton T, Blubaugh I, Pauli J et al. Paging “Dr. Google”: does technology fill the gap created by the prenatal care visit structure? Qualitative focus group study with pregnant women. Journal of Medical Internet Research. 2014;16(6): e147.doi.10.2196/jmir.3385. 10.2196/jmir.3385 PMC4060145 24892583

[39] Asiodu IV, Waters CM, Dailey DE, Lee KA, Lyndon A. Breastfeeding and use of social media among first-time African American mothers. Journal of Obstetric, Gynecologic, & Neonatal Nursing. 2015;44(2):268–278. doi.10.1111/1552-6909.12552. 10.1111/1552-6909.12552 PMC4359664 25712127

[40] Langarizadeh M, Behzadian H, Samimi M (2016). Development of personal health record application for gestational diabetes, based on smart phone. The J Urmia Nurs Midwifery Fac.

[41] Tahamtan I, Sedghi S (2012). The use of personal digital assistants and smartphones to access health information. J Payavard Salamat.

[42] Mousavi Chalac A, Riahi A. Information Needs of Pregnant Women Referred to Health Centers in Behshahr City during 2016-17. Journal of Community Health Research. 2017;6(3):165 – 74. URL: http://jhr.ssu.ac.ir/article-1-375-en.html.

[43] Shieh C, Mays R, McDaniel A, Yu J. Health Literacy and Its Association With the Use of Information Sources and With Barriers to Information Seeking in Clinic-Based Pregnant Women. Health Care for Women International. 2009;30(11):971 – 88. doi.10.1080/07399330903052152. 10.1080/07399330903052152 19809901

[44] Sayakhot P, Carolan-Olah M. Internet use by pregnant women seeking pregnancy-related information: a systematic review. BMC Pregnancy and Childbirth. 2016;16(1):65. doi.10.1186/s12884-016-0856-5. 10.1186/s12884-016-0856-5 PMC4810511 27021727

[45] Song H, Cramer EM, McRoy S, May A, Information Needs. Seeking Behaviors, and Support Among Low-Income Expectant Women. Women & Health. 2013;53(8):824 – 42. doi.10.1080/03630242.2013.831019. 10.1080/03630242.2013.831019 24215275

