# Supplementary material for: Pregnant women’s use and attitude toward Mobile phone features for self-management
Source: BMC Med Inform Decis Mak. 2023 Apr 26;23:77. doi: 10.1186/s12911-023-02172-w (PMC10134552; doi:10.1186/s12911-023-02172-w)
Supplement: Supplementary file 1 — Supplementary Material 1 [file 12911_2023_2172_MOESM1_ESM.docx]

Additional Fail 1: The frequency of use and attitude to use mobile phone for receiving prenatal care services (N=158)

| **Item** | | **None** | **Phone/Voice Call** | **SMS** | **Internet Search** | **Social Media** | **Email** | **Software/Apps** | **Video Call** |
| --- | --- | --- | --- | --- | --- | --- | --- | --- | --- |
| **Use** | Receiving information about nutritional needs (useful and harmful food | 56(35.4) | 36(22.8) | 11(7) | 54(32.4) | 47(29.7) | 0 | 25(15.8) | 2(1.3) |
|  | Receiving information about exercise and fitness (useful and harmful activities) | 79(50) | 7(4.4) | 2(1.3) | 40(25.3) | (21.5)34 | 1(0.6) | (8.2)13 | 2(1.3) |
|  | Receiving information about pregnancy complications and types of deliveries | 55(34.8) | 15(9.5) | 2(1.3) | (35.4)56 | (22.6)42 | 1(0.6) | (9.5)15 | 1(0.6) |
|  | Receiving information about changes in pregnancy (fetal development) | 58(36.7) | 15(9.5) | 7(4.4) | (36.1)57 | (22.6)42 | 2(1.3) | (14.6)23 | 1(0.6) |
|  | Receiving information about taking harmful drugs medications during pregnancy | 51(32.3) | 21(13.3) | 5(3.2) | (42.4)67 | (22.8)36 | 1(0.6) | (9.5)15 | 2(1.3) |
|  | Receiving information about mental health issues (such as depression) | (49.4)78 | 9(5.7) | 5(3.2) | (27.5)43 | (20.3)32 | 1(0.6) | (8.2)13 | 2(1.3) |
|  | Receiving information about vaccination | (51.9)82 | 21(13.3) | (3.8)6 | (21.5)34 | (17.8)28 | 1(0.6) | (6.3)10 | 2(1.3) |
|  | Communicating with other pregnant women | (59.5)94 | (21.5)34 | (6.3)10 |  | (19.6)31 | 0 | 2(1.3) | 2(1.3) |
|  | Reminders about time appointments with physician | (67.1)106 | (20.9)33 | (6.3)10 |  | (7.6)12 | 0 | 3(1.9) | 3(1.9) |
|  | Reminders about taking drugs medications and dietary supplements | (77.8)123 | (8.9)14 | (4.4)7 |  | (7)11 | 0 | 5(3.2) | (2.5)4 |
|  | Reminders about doing lab tests | (70.9)112 | (15.8)25 | (3.8)6 |  | (6.3)10 | 0 | 5(3.2) | (1.9)3 |
|  | Reminders about doing ultrasound | (70.9)112 | (16.5)26 | (3.2)5 |  | (7.6)12 | 0 | 5(3.2) | (1.9)3 |
| **Attitude** | Receiving information about nutritional needs (useful and harmful food | (32.3)51 | (11.4)18 | (6.3)10 | (32.9)52 | (35.5)53 | 2(1.3) | (8.9)14 | 2(1.3) |
|  | Receiving information about exercise and fitness (useful and harmful activities) | (38)60 | (9.5)15 | (7)11 | (27.8)44 | (32.3)51 | 2(1.3) | (6.3)10 | 2(1.3) |
|  | Receiving information about pregnancy complications and types of deliveries | (34.8)55 | (10.8)17 | (4.4)7 | (31)49 | (31.6)50 | 2(1.3) | (7.6)12 | 2(1.3) |
|  | Receiving information about changes in pregnancy (fetal development) | (29.7)47 | (13.9)22 | (4.4)7 | (31)49 | (32.3)51 | 2(1.3) | (10.8)17 | 2(1.3) |
|  | Receiving information about taking harmful drugs medications during pregnancy | (34.2)54 | (13.9)22 | (5.7)9 | (27.8)44 | (31)49 | 1(0.6) | (8.9)14 | 2(1.3) |
|  | Receiving information about mental health issues (such as depression) | (41.8)66 | (10.8)17 | (5.7)9 | (25.3)40 | (27.2)43 | 1(0.6) | (6.3)10 | 2(1.3) |
|  | Receiving information about vaccination | (39.9)63 | (17.7)28 | (7.6)12 | (22.2)35 | (22.6)42 | 2(1.3) | (6.3)10 | 2(1.3) |
|  | Communicating with other pregnant women | (49.4)78 | (19.6)31 | (7.6)12 |  | (29.1)46 | 0 | 5(3.2) | (2.5)4 |
|  | Reminders about time appointments with physician | (55.7)88 | (19.6)31 | (9.5)15 |  | (19)30 | 1(0.6) | (5.1)8 | 2(1.3) |
|  | Reminders about taking drugs medications and dietary supplements | (58.2)99 | (17.7)28 | (10.8)17 |  | (15.2)24 | 0 | (6.3)10 | 2(1.3) |
|  | Reminders about doing lab tests | (56.3)88 | (18.4)29 | (10.1)16 |  | (16.5)26 | 0 | (5.1)8 | (1.9)3 |
|  | Reminders about doing ultrasound | (56.3)89 | (19)30 | (10.1)16 |  | (16.5)26 | 1(0.6) | (4.4)7 | 2(1.3) |

Additional files 2: The frequency of use and attitude to use mobile phone in pregnant women in terms of demographic details (N=158)

| **Demographic Variable** | | **Use** | | | | **Attitude** | | | |
| --- | --- | --- | --- | --- | --- | --- | --- | --- | --- |
|  |  | **Low** | **Moderate** | **High** | **P-value** | **Low** | **Moderate** | **High** | **P-value** |
| **Age (years)** | **25 and younger** | 12 (23.1) | 26 (50) | 14 (26.9) | 0.885 | 35 (67.3) | 1 (1.9) | 16 (30.8) | 0.355 |
|  | **26 and above** | 27 (25.5) | 54 (50.9) | 25 (23.6) |  | 78 (73.6) | 5 (4.7) | 23 (21.7) |  |
| **Education level** | **Less than bachelor's degree** | 31 (33.3) | 45 (48.4) | 17 (18.3) | 0.004 | 75 (80.6) | 4 (4.3) | 14 (15.1) | 0.004 |
|  | **Higher than bachelor's degree** | 8 (12.3) | 35 (53.8) | 22 (33.8) |  | 38 (58.5) | 2 (3.1) | 25 (38.5) |  |
| **Area of residence** | **City** | 36 (23.7) | 77 (50.7) | 39 (25.7) | 0.206 | 107 (70.4) | 6 (3.9) | 39 (25.7) | 0.289 |
|  | **Village** | 3 (50) | 3 (50) | 0 |  | 6 (100) | 0 | 0 |  |
| **Under supervision** | **Physician** | 33 (23.2) | 74 (52.1) | 35 (24.6) | 0.872 | 103 (72.5) | 5 (3.5) | 34 (23.9) | 0.637 |
|  | **Midwife** | 2 (40) | 2 (40) | 1 (20) |  | 4 (80) | 0 | 1 (20) |  |
|  | **Health center** | 3 (42.9) | 2 (28.6) | 2 (28.6) |  | 3 (42.9) | 1 (14.3) | 3 (42.9) |  |
|  | **Without supervision** | 1 (25) | 2 (50) | 1 (25) |  | 3 (75) | 0 | 1 (25) |  |
| **Nationality** | **Iranian** | 35 (23) | 79 (52) | 38 (25) | 0.049 | 108 (71.1) | 5 (3.3) | 39 (25.7) | 0.112 |
|  | **Non-Iranian** | 4 (66.7) | 1 (16.7) | 1 (16.7) |  | 5 (83.3) | 1 (16.7) | 0 |  |
| **Employed** | **Yes** | 4 (12.1) | 16 (48.5) | 13 (39.4) | 0.042 | 20 (60.6) | 1 (3) | 12 (36.4) | 0.216 |
|  | **No** | 35 (28) | 64 (51.2) | 2 (20.8) |  | 93 (74.4) | 5 (4) | 27 (21.6) |  |
| **Pregnancy month** | **5 and less** | 21 (27.6) | 34 (44.7) | 21 (27.6) | 0.361 | 51 (67.1) | 2 (2.6) | 23 (30.3) | 0.25 |
|  | **6 and above** | 18 (22) | 46 (56.1) | 18 (22) |  | 62 (75.6) | 4 (4.9) | 16 (19.5) |  |
| **Previous pregnancy** | **Yes** | 23 (30.3) | 45 (59.2) | 8 (10.5) | 0.001 | 50 (61) | 3 (3.7) | 29 (35.4) | 0.005 |
|  | **No** | 16 (19.5) | 35 (42.7) | 31 (37.8) |  | 63 (82.9) | 3 (3.9) | 10 (13.2) |  |
